# Supplementary material for: Machine learning in spectral domain
Source: Nat Commun. 2021 Feb 26;12:1330. doi: 10.1038/s41467-021-21481-0 (PMC7910623; doi:10.1038/s41467-021-21481-0)
Supplement: Supplementary file 1 — Supplementary Information [file 41467_2021_21481_MOESM1_ESM.pdf]

Supplementary material – Machine learning in spectral domain.

Lorenzo Giambagli[1], Lorenzo Buffoni[1,2],
Timoteo Carletti[3], Walter Nocentini[1],
Duccio Fanelli[1]

1. *Università degli Studi di Firenze, Dipartimento di Fisica e Astronomia, CSDC and INFN, via G. Sansone 1, 50019 Sesto Fiorentino, Italy*
2. *Dipartimento di Ingegneria dell'Informazione, Università di Firenze, Via S. Marta 3, 50139 Florence, Italy and*
3. *naXys, Namur Institute for Complex Systems, University of Namur, 8 Rempart de la Vierge, B5000 Namur, Belgium*

SUPPLEMENTARY NOTE 1

In this Supplementary Material we will apply the spectral learning technique for a different dataset, the so called Fashion MNIST. In the following we shall introduce the specificity of the employed dataset and then turn to illustrate the results of the analysis.

Fashion MNIST

Fashion MNIST is a dataset composed of Zalando's articles images. It is made of 60000 images as train set and 10.000 images as test set. Each example is a 28×28 pixels image with greyscale color shading. Every element in the training and test set is assigned one label as detailed in Table I.

We shall hereafter report on the results obtained when applying the spectral learning technique to the Fashion MNIST dataset.

Linear and nonlinear spectral learning applied to Fashion MNIST

Figure 1 shows the results obtained when adopting the *linear wide* configuration, as it was named in the main body of the paper. More specifically, we consider an architecture composed of three nested layers. The first and the last are respectively made of $N_1 = 784$ and $N_3 = 10$ nodes. The size of the second layer is varied at will. In the main panel of Figure 1, the accuracy of the wide learning scheme is plotted as a function of $N_2 + N_3$. The red line refers to the simplified scheme where (a subset of) the eigenvalues are solely tuned (while leaving the eigenvectors fixed at the random realization set by the initial condition). The blue line stands for the accuracy of the neural network trained in direct space. In analogy with what reported in the main body of the paper, the target of the optimization is a subset of cardinality $N_2 + N_3$ of the $N_1N_2 + N_2N_3$ weights which could be in principle trained in the space of the nodes. The performance obtained when training via the spectral approach are clearly superior, in line with what reported in the main body of the paper. The black line displays the accuracy of the linear neural network when acting, in direct space, on the full set of $N_1N_2 + N_2N_3$ trainable parameters.

In Figure 2 the results for the *deep linear* configuration are reported. Here, the entry layer is made of $N_1 = 784$ elements and the output one has $N_\ell = 10$ nodes. The size of the second layer is set to $N_2 = 800$. The first symbol in the series refer to $\ell = 3$. The following three points of the collection are obtained by setting $\ell = 4$, $N_2 = 800$ and letting N_3 to take values $(100, 300, 600)$.

The last few points refer to $\ell = 5$, $N_2 = 800$, $N_3 = 600$ and $N_4 = (100, 300, 500)$. Symbols are analogous to those employed in Figure 1. Here again, spectral learning yields better performance.

Finally, the results for a specific non linear architecture are displayed Figure 3. We have here chosen to deal with an activation function of the *Relu* type. The size of the second layer is varied and the accuracy of the classifier computed. As for a fully linear architecture, by adjusting the eigenvalues yields better classification performances as compared to those obtained when operating in direct space, with an identical number of trainable parameters (randomly selected from the pool of tunable weights). The green line refer to the spectral learning when eigenvalues and eigenvectors are simultaneously trained

Table ‘II summarizes the obtained results. We report in particular the best (average) performance of the different training schemes for distinct architectures and over the explored range of sizes.

SUPPLEMENTARY FIGURES

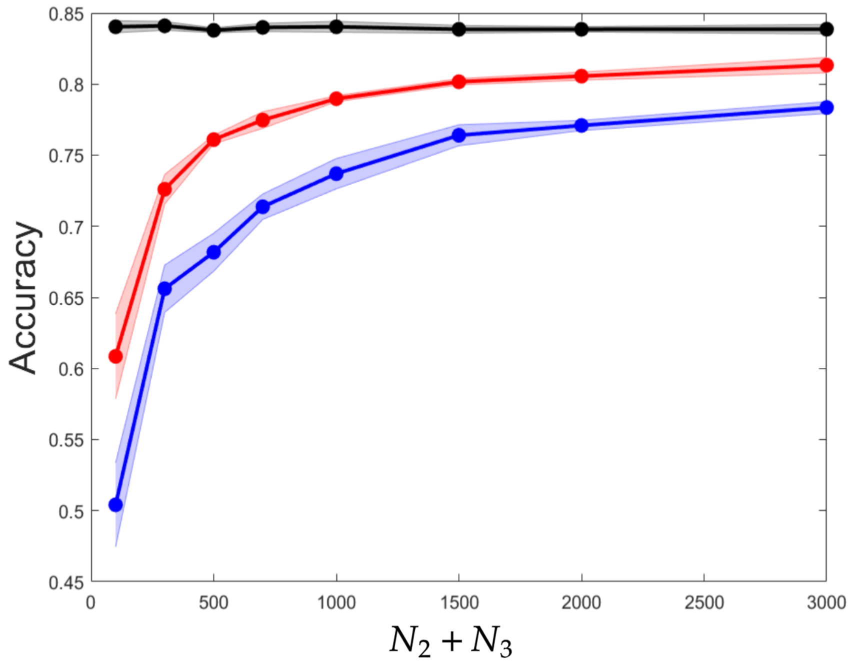

Supplementary Fig. 1. The red line reports on the performance of the spectral training. The blue line refers to the neural network trained in direct space optimizing $N_2 + N_3$ parameters, a subset of the total number of adjustable weights $N_1 N_2 + N_2 N_3$. The black line stands for the accuracy displayed when training the full set of available parameters direct space.

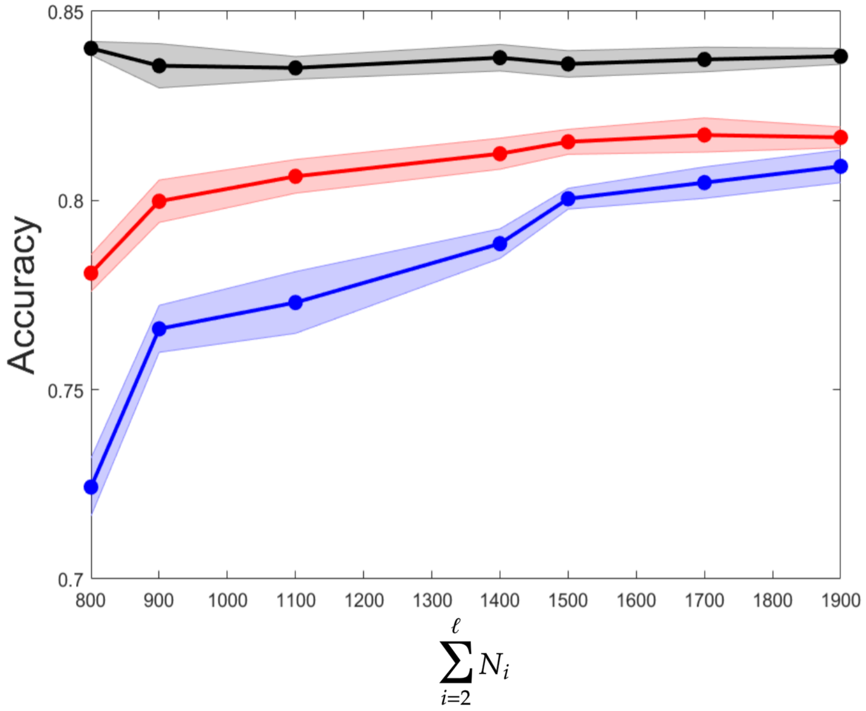

Supplementary Fig. 2. The accuracy obtained for a *deep linear* architecture is plotted against the number of trainable parameters. Symbols are chosen as in Figure 1. The size of the second layer is chosen to be $N_2 = 800$ and the first points of the collection refer to a simple three layer configuration. The following three points are obtained for $\ell = 4$, $N_2 = 800$ and $N_3 = (100, 300, 600)$. The last points refer to $\ell = 5$, $N_2 = 800$, $N_3 = 600$ and $N_4 = (100, 300, 500)$.

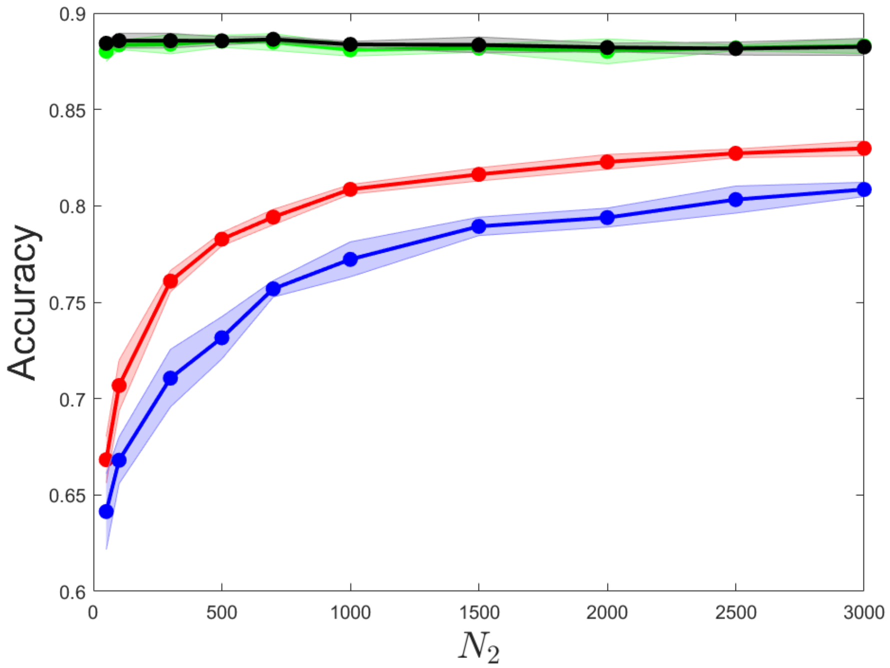

Supplementary Fig. 3. The accuracy of the non-linear deep neural network is plotted varying N_2, the size of the second linear layer. As for the MNIST dataset, we assume a four layers $\ell = 4$ network $N_3 = 130$. The non linearity associated to this latter layer is of the *ReLu* type. The red line refers to the spectral training, with $N_2 + N_3 + N_4$ adjusted eigenvalues. The blue line refers to the results obtained for an equivalent deep neural network, when training a subset of (randomly selected) N_i parameters per layer i, with $i > 1$. The black line reports the accuracy of the neural network when training the full set of weights. The green line refer to the spectral learning when simultaneously training eigenvalues and eigenvectors.

SUPPLEMENTARY TABLES

**Label**	**Description**
0	T-shirt/top
1	Trouser
2	Pullover
3	Dress
4	Coat
5	Sandal
6	Shirt
7	Sneaker
8	Bag
9	Ankle boot

Supplementary Table I. The characteristic features of Fashion-MNIST database are listed.

**F-MNIST best mean accuracy over the explored range**	*linear wide*	*linear deep*	*non linear*
Constrained NN	79.8 %	81.8 %	80.7 %
Train Λ	81.7 %	82.4 %	83.1 %
Unconstrained NN	83.7 %	83.4 %	88.8 %
Train Λ, Φ	84.1 %	84.0 %	89.4 %

Supplementary Table II. The best (average) performance of the different training schemes are displayed.
