# Supplementary material for: Machine learning in spectral domain
Source: Nat Commun. 2021 Feb 26;12:1330. doi: 10.1038/s41467-021-21481-0 (PMC7910623; doi:10.1038/s41467-021-21481-0)
Supplement: Supplementary file 2 — Peer Review File [file 41467_2021_21481_MOESM2_ESM.pdf]

Reviewer #1 (Remarks to the Author):

The method proposed seems to be truly novel and that makes this an area worthy of study. However, the results achieved at this time do not seem very significant. If they are, and the reviewer is mistaken, this needs to be brought out in the paper better and using direct comparisons to some baseline and exact numbers. Examples of inexact terms include "up to about 98%". This is especially relevant in the context of MNIST which is generally considered to be nearly solved, and thus fractions of a percentage are important for distinction between methods.

Regarding the abstract:
"Further, it is surmised that the nested indentation of eigenvectors that defines the core idea of spectral learning could help understanding why deep networks work as well as they do." This statement does not seem to be addressed in the body of the paper.

Regarding the introduction section:
In the introduction, the authors use the term AI four times. The second and third time, the usage is entirely appropriate. However, the first time it is referenced, the authors state "The core idea, common to all artificial intelligence (AI) technology, is that systems can learn from data…" When an algorithm learns from data that makes it properly a machine learning algorithm, which is a subset of AI. AI is a broader field and includes algorithms that are independent of and do not improve with data. The fourth time the authors use the term, they are speaking about neural networks and it might be prudent just to entirely eliminate the following clause: "which define the skeleton of modern AI architectures."

In the second paragraph of the introduction, the authors speak in terms of conditioning, reward, and punishment. These terms are generally more appropriately applied in reinforcement learning, which is machine learning, but the reinforcement learning algorithms are distinct from the neural network algorithms, even when used in combination in advanced models such as Google's AlphaGo. Although conceptually similar, in neural network training, terminology like conditioning, reward and punishment is not common, and instead is usually simply referred to as minimizing the classification error.

In the fourth paragraph of the introduction, the authors use the term "recursively" when they mean "iteratively".

"We speculate that this is a universal paradigm of learning which holds in general beyond the specific setting here explored." This bold statement should be at least somewhat justified.

Regarding the results section:
The second section of the paper, titled "Results", forms the bulk of the paper, and it took this reviewer several passes to gain an understanding of what was being presented here. The reviewer suggests reorganizing this into several sections following the general pattern of other research papers in the field. For example, it would be prudent to first describe the new method in its own section. Then, moving on to a separate section that details the experiments performed.

General comments:
The primary weakness of this paper is the lack of comparison with other methods. The paper does mention the error achieved by a simple linear classifier and the current state-of-the-art results on the MNIST dataset. The problem comes when statements like the following are made: "The performance are competitive with standard schemes…", "These latter display competitive performance as compared to standard deep learning schemes…", "…while returning competitive performance scores…", "This is a

competitive figure as compared to standard ML techniques", "…we reach performances which are almost identical, within statistical errors, to those obtained with usual training protocols", and "performances which are comparable to those obtained with (the corresponding) NN trained with standard algorithms." These statements are made without providing actual values, citations, and/or results from baseline experiments. The authors could improve this paper considerably by providing a more systematic approach to the experimentation and presentation of those results. Tables with exact values of experimental outcomes would be beneficial, including values for either baseline experiments or values in additional published research. The following paper, chosen here because it is relatively easy to understand and in the field of machine learning, is a good example of thorough experimentation and presentation of those results: https://ieeexplore.ieee.org/stamp/stamp.jsp?tp=&arnumber=7898482. This paper is also a good example of a well-structured paper with distinct sections serving specific purposes. (The reviewer is not one of, nor does he have a relationship with any of the authors of this specific paper—he has simply read it recently and it came to mind as a good example.)

The authors would be well advised to propose a motivation for this new method. Based on several comments throughout the paper, it would seem to be that minimizing the number of parameters needed by a model to achieve a certain level of accuracy is a candidate. If this is the case, the authors should include results for baseline and/or alternate architectures using a similar number of parameters as a means of demonstrating the superiority of their method. The authors would additionally be well advised to address the reasons behind not using convolution, the fundamental operation used by all neural networks for image classification methods currently in use.

Occasional very minor grammatical errors that could be improved by employing the use of a native English-speaking editor. Examples:
- "These latter underlies however an highly…". "underlies" suggests a singular subject and should agree in number with the subject "these", which is plural.
- "The performance are…". Another subject/verb number agreement problem.
- There are several more subject/verb number agreement problems.
- In most cases of the word "non" in the paper, it should either be directly added to the following word or joined to the following word with a hyphen.

The authors included a reference to source code that could be used to reproduce the results, and this is lauded by the reviewer. The reviewer downloaded the code and ran the experiment in it locally and attained comparable results. The reviewer used 20 epochs for all of the following mentioned experiments. With five trials, the reviewer received a 96.804% (+/- 0.094) mean accuracy using the code published by the authors.

The batch size of 5,000 is unusually large, so the reviewer repeated the experiment with a batch size of 100 and achieved a 97.524% (+/- 0.044) mean accuracy. The authors would be advised to use the smaller batch size in the revision to attain better results.

The reviewer chose to implement a potential baseline experiment by replacing the spectral layers with traditional fully connected layers with batch normalization and ReLU activation (this is the current standard/common practice). The reviewer used the same number of layers (3) and chose the neurons so that the network had a nearly identical number of trainable parameters. The authors can replace these lines in their source code:

```
model.add(SpectralLayer(500,is_base_trainable=False,activation='tanh'))
model.add(SpectralLayer(200,is_base_trainable=True,activation='tanh'))
model.add(SpectralLayer(10,is_base_trainable=True,activation='softmax'))
```

with these to get the same baseline:

```
model.add(tf.keras.layers.Dense(120))
model.add(tf.keras.layers.BatchNormalization())
model.add(tf.keras.layers.Activation('relu'))
```

```
model.add(tf.keras.layers.Dense(60))
model.add(tf.keras.layers.BatchNormalization())
model.add(tf.keras.layers.Activation('relu'))
model.add(tf.keras.layers.Dense(10, activation='softmax'))
```
When using a batch size of 5,000, this baseline achieved a 97.054% (+/- 0.161) mean accuracy. With a batch size of 100, a 98.058% (+/- 0.085) mean accuracy was achieved.

The reviewer then went on to experiment with a 3-layer convolutional neural network with a similar number of parameters. The relevant code follows (the shaping of the input data is omitted):
```
model.add(tf.keras.layers.Conv2D(12, (3, 3)))
model.add(tf.keras.layers.BatchNormalization())
model.add(tf.keras.layers.Activation('relu'))
model.add(tf.keras.layers.Conv2D(16, (3, 3)))
model.add(tf.keras.layers.BatchNormalization())
model.add(tf.keras.layers.Activation('relu'))
model.add(tf.keras.layers.Conv2D(20, (3, 3)))
model.add(tf.keras.layers.BatchNormalization())
model.add(tf.keras.layers.Activation('relu'))
model.add(tf.keras.layers.Flatten())
model.add(tf.keras.layers.Dense(10, activation='softmax'))
```
When using a batch size of 100, this achieved a 98.826% (+/- 0.148) mean accuracy.
The authors would be well advised to put forward such baselines and work towards showing their method as superior.

The reviewer, Adam Byerly, wishes the authors well and looks forward to the future direction of their work.

Reviewer #4 (Remarks to the Author):

The manuscript "Machine learning in spectral domain" is sound and mostly well written.
Although it contains some interesting ideas,
I fail to see any particularly novel result or strong conceptual/computational advance supporting its publication on Nat. Comm.
Also, comparison with related literature and benchmarking against models therein is missing,
as is also missing an in-depth assessment of the performance of the proposed model.
As the paper currently stands, the authors fail to substantiate the claim that the proposed paradigm is a promising alternative for deep learning practice or theory,
but I do not exclude the possibility of changing my mind if much stronger evidence is provided.

My major concerns are the following:

- This is easily fixed, but caused me some major pain in understanding the paper: it is not made explicit how to construct vec{n} beyond the first N_1 components. I assume the last N-N1 components to be just set to 0, but really, this should be clearly stated.
The same goes for the entries of the base vectors \phi_k, which I assume to take value 0 except for the diagonal and the sub-diagonal blocks.

- The framework boils down to a standard deep network where each layer performs a linear transformation of the input, possibly followed by an elementwise non-linearity.
The only difference with the standard approach is in the structured form of the weight matrices, depending quadratically on the eigenvectors phi and linearly on the eigenvalues lambda. The number of training parameters is reduced only when fixing the vectors phi, with subsequent severe

performance impact. No time or memory gain is obtained in either case (actually, it could quite be the contrary).
I can think of a few approaches that involve learning structured matrices, some of which also lead to time and space complexity gains:
- separable convolutions
- low-rank factorizations, as in
"Low-rank matrix factorization for Deep Neural Network training with high-dimensional output targets", DOI:10.1109/ICASSP.2013.6638949
- training of singular values of the SVD decomposition only, as discussed in
"Entropy and mutual information in models of deep neural networks" DOI:10.1088/1742-5468/ab3430
- porcupine neural networks and related models:
https://arxiv.org/abs/1710.02196
https://arxiv.org/abs/2003.00152

None of these approaches is discussed in the manuscript, let alone compared and benchmarked against the proposed model.

- When also training the eigenvectors ϕ_k, the only reduction in the number of trained parameters comes from freezing the first layer.
In this setting, performance should be compared against standard deep networks with the same number of trained and untrained parameters where the first layer is frozen.
In fact, the claim "Learning on the eigenvalues and eigenvectors allows to significantly reduce the num-
ber of free parameters usually managed in conventional neural networks" should be validated comparing with standard architectures with the same number of trained AND untrained parameters, while only a fully trained standard network with no hidden layers was used for the result in fig. 3.
As a side note, since this setting is also referred to as "extreme learning", the authors should mention and comment on this.

When training only lambdas instead, it would be interesting to see a comparison with the technique of https://arxiv.org/abs/2003.00152
or
"Entropy and mutual information in models of deep neural networks" DOI:10.1088/1742-5468/ab3430

- Practical implementation and computational complexity of the algorithm are not clearly stated.
I assume that a reasonable implementation of a spectral layer involves multiplication with $(N_k + N_{k+1}) \times (N_k + N_{k+1})$ matrices in the forward pass, so roughly twice the size of a corresponding standard layer.
Could this be brought down to multiplication of $N_k \times N_{k+1}$ matrices exploiting (again) the block structure?

- I may be wrong, but I don't think that statements as "It is in fact tempting to surmise that the nested arrangement of the eigenvectors of the adjacency matrix which define the network in direct space is a key property for the neural network to carry out the assigned classification task." or "By formulating the learning process in reciprocal space, we have isolated an important aspect which, we believe, could form the basis for a rational understanding of the surprising ability of deep networks to cope with the assigned tasks" have been really substantiated, and that the so-called "nested structure" brings any more understanding or is conceptually any different from the plain concept of forward propagation.

- Number of parameters in ordinary schemes do not necessarily ramp up as N^2 as stated in the conclusions, there is an interplay between depth d and width w,
number of parameters being $w^2 * d$ and $N = w*d$.

Answer to Reviewer #1

The method proposed seems to be truly novel and that makes this an area worthy of study. However, the results achieved at this time do not seem very significant. If they are, and the reviewer is mistaken, this needs to be brought out in the paper better and using direct comparisons to some baseline and exact numbers. Examples of inexact terms include "up to about 98%". This is especially relevant in the context of MNIST which is generally considered to be nearly solved, and thus fractions of a percentage are important for distinction between methods.

We thank the referee, Dr. Adam Byerly, for the very many relevant observations that he raised. We really appreciate it a lot and we have seriously worked so as to account for his remarks. As stated in the letter to the Editor, we are sincerely convinced that carrying out the training in reciprocal space opens up novel perspectives to supervised learning. In the revised version of the manuscript, we now perform several additional tests to provide convincing evidence in this direction. In particular, we now show that by adjusting the eigenvalues, when freezing the eigenvectors entries, yields performances which are superior to those attained with usual algorithms restricted to operate with an identical number of free parameters. Further, we show that spectral learning limited to the eigenvalues returns a distribution of the predicted weights which is close to that obtained when training the neural network in direct space, with no restrictions on the parameters to be tuned. Spectral learning bound to the eigenvalues could be hence employed for pre-training of deep neural networks, in conjunction with conventional machine learning schemes. In the revised version of the paper we have explored different configurations (including the simultaneous learning of eigenvalues and eigenvectors). The obtained results are displayed in a novel set of figures which we hope will help to clarify the overall message. In this respect, it is not our goal to come up with an architecture or a learning scheme which contributes to solving the NMIST problem beyond current limits. The NMIST database has been selected because of its inherent simplicity as the ideal playground to illustrate the ideas underlying the concept of spectral learning. This is now acknowledged in the revised version of the manuscript.

Regarding the abstract:
"Further, it is surmised that the nested indentation of eigenvectors that defines the core idea of spectral learning could help understanding why deep networks work as well as they do." This statement does not seem to be addressed in the body of the paper.

The sentence has been removed from the abstract (which has been entirely rewritten). The idea that we have in mind is however the following. Working in

the reciprocal space, we show that the information is passed between mutually entangled eigendirections. For a feed-forward architecture in direct space, the indentation (as we call it) of the eigenvectors belonging to different processing units is rather obvious. The method that we propose is however not limited to the explored setting. It can be in fact straightforwardly generalized to other contexts, the only requirement being the assumption of a non orthogonal basis (hence indented, in the introduced jargon) in reciprocal space. For this reason, it was speculated, in the previous version of the manuscript, that the nested indentation is a universal paradigm. We agree with the referee that this was not sufficiently motivated. In the revised version of the paper we discuss some aspects related to this possibility, emphasising the role of non-orthogonality.

Regarding the introduction section:
In the introduction, the authors use the term AI four times. The second and third time, the usage is entirely appropriate. However, the first time it is referenced, the authors state "The core idea, common to all artificial intelligence (AI) technology, is that systems can learn from data…" When an algorithm learns from data that makes it properly a machine learning algorithm, which is a subset of AI. AI is a broader field and includes algorithms that are independent of and do not improve with data. The fourth time the authors use the term, they are speaking about neural networks and it might be prudent just to entirely eliminate the following clause: "which define the skeleton of modern AI architectures."

We thank the referee for pointing this out. We have modified the introduction accordingly.

In the second paragraph of the introduction, the authors speak in terms of conditioning, reward, and punishment. These terms are generally more appropriately applied in reinforcement learning, which is machine learning, but the reinforcement learning algorithms are distinct from the neural network algorithms, even when used in combination in advanced models such as Google's AlphaGo. Although conceptually similar, in neural network training, terminology like conditioning, reward and punishment is not common, and instead is usually simply referred to as minimizing the classification error.

We agree with the referee and we have modified the text accordingly.

In the fourth paragraph of the introduction, the authors use the term "recursively" when they mean "iteratively".

This is correct. We have amended the text accordingly.

“We speculate that this is a universal paradigm of learning which holds in general beyond the specific setting here explored.” This bold statement should be at least somewhat justified.

The sentence has been removed. We are confident that, in the revised version of the manuscript, our claims are better substantiated (by the new numerical results therein reported).

Regarding the results section:
The second section of the paper, titled “Results”, forms the bulk of the paper, and it took this reviewer several passes to gain an understanding of what was being presented here. The reviewer suggests reorganizing this into several sections following the general pattern of other research papers in the field. For example, it would be prudent to first describe the new method in its own section. Then, moving on to a separate section that details the experiments performed.

Following the referee’s remark, we have restructured the paper completely. Right after the introduction, in a section termed “Linear and nonlinear spectral learning”, we now introduce the mathematical formalism and discuss the specificity of the proposed method. We have also rewritten from scratch the part devoted to explaining the linear version of the method and worked to better integrate linear and non linear frameworks. The results, i.e. the application of the method to the MNIST database, are presented in a self-contained section. The numerical tests have been considerably extended, as compared to the earlier version of the manuscript.

General comments:
The primary weakness of this paper is the lack of comparison with other methods. The paper does mention the error achieved by a simple linear classifier and the current state-of-the-art results on the MNIST dataset. The problem comes when statements like the following are made: “The performance are competitive with standard schemes…”, “These latter display competitive performance as compared to standard deep learning schemes…”, “…while returning competitive performance scores…”, “This is a competitive figure as compared to standard ML techniques”, “…we reach performances which are almost identical, within statistical errors, to those obtained with usual training protocols”, and “performances which are comparable to those obtained with (the corresponding) NN trained with standard algorithms.” These statements are made without providing actual values, citations, and/or results from baseline experiments. The authors could improve this paper considerably by providing a more systematic approach to the experimentation and presentation of those results. Tables with exact values of experimental

outcomes would be beneficial, including values for either baseline experiments or values in additional published research.

As discussed above, it is not our intention to challenge state-of-the-art implementations in the competition for the best classification performance of the MNIST database. We have no technical resources and/or competences for achieving this goal. Our objective is instead to propose a proof-of-concept by discussing and analysing a novel route to supervised learning, which, to the best of our knowledge, has not been considered so far in the literature. We however agree with the referee that several statements made in the former version of the manuscript lacked the necessary backup and we have worked on the text to improve on this. More specifically, we now draw a systematic comparison between the results respectively obtained for (i) spectral learning targeted to (a subset of) the eigenvalues (while freezing the eigenvectors) and (ii) learning in the space of the nodes (also called direct space) restricted to a set of randomly selected weights of identical cardinality (of the set of adjusted eigenvalues). The performance of the spectral method proves clearly superior, as it can be readily appreciated by visual inspection of Fig. 5, 6, 7. It hence turns out that eigenvalues define a relatively small set of parameters of paramount importance for neural networks training, way more strategic than any other set of equivalent cardinality in the space of the nodes. As such, they allow for a global approach to the learning, with significant reflexes of fundamental and applied interest. This is at variance with standard approaches which seek at implementing local modifications in the space of the nodes, as we now discuss in the revised version of the manuscript. This is one of our main conclusions which is also confirmed by inspecting the distribution of the weights in the space of the nodes, as obtained when training the neural network in the spectral domain. Performing the training on the eigenvalues returns a distribution of the weights that looks quite similar to that obtained when training the neural network in direct space, by using the full set of available parameters. This result hints again to the global character of the learning anchored to dual space. Extending the training so as to include the eigenvectors' entries in the set of trained parameters yields a substantial improvement of the performance score (see Fig. 7). A large part of the discussion is devoted however to a simplified setting where eigenvalues are solely optimized so as to hopefully communicate the main ideas that inspire the newly proposed technique.

The following paper, chosen here because it is relatively easy to understand and in the field of machine learning, is a good example of thorough experimentation and presentation of those results: https://ieeexplore.ieee.org/stamp/stamp.jsp?tp=&arnumber=7898482. This paper is also a good example of a well-structured paper with distinct sections serving specific purposes. (The reviewer is not one of, nor does he have a

relationship with any of the authors of this specific paper—he has simply read it recently and it came to mind as a good example.)

We thank the referee for suggesting this work. We took some inspiration from the way the paper is organized.

The authors would be well advised to propose a motivation for this new method. Based on several comments throughout the paper, it would seem to be that minimizing the number of parameters needed by a model to achieve a certain level of accuracy is a candidate. If this is the case, the authors should include results for baseline and/or alternate architectures using a similar number of parameters as a means of demonstrating the superiority of their method. The authors would additionally be well advised to address the reasons behind not using convolution, the fundamental operation used by all neural networks for image classification methods currently in use.

We have revised the paper to hopefully make our main message more transparent. As mentioned earlier, the central idea is that neural networks can be trained by using a radically different strategy. The target of the learning are the spectral properties of the transfer operators, in direct space. This is not an innocent shift, in our opinion. On the contrary, it comes with several non trivial implications that we try to substantiate in the revised version of the manuscript. This includes approaching the learning from a global perspective, as opposed to the local optimization that is deep-rooted in standard approaches. Starting from these premises, we elaborate in various directions to bring into evidence some of the advantages of the introduced method. There are surely other directions that could be possibly explored to critically challenge the ability of the method and we hope to raise the interest of professional machine learning scientists to contribute in this sense. When it comes to convolution, we believe that the method can be easily extended to embrace other, more complex, settings. In this first work dedicated to the topic we have however decided to operate in the simplest possible scenario (to our understanding) to set the stage for the spectral learning protocol, and, at the same time, minimize other complications of technical nature. Following the suggestion of the referee, in all tests performed we made comparison with the results obtained with baseline architectures operated with a similar number of parameters.

Occasional very minor grammatical errors that could be improved by employing the use of a native English-speaking editor. Examples:
- “These latter underlies however an highly…”. “underlies” suggests a singular subject and should agree in number with the subject “these”, which is plural.
- “The performance are…”. Another subject/verb number agreement problem.
- There are several more subject/verb number agreement problems.

- In most cases of the word “non” in the paper, it should either be directly added to the following word or joined to the following word with a hyphen.

We thank the referee for spotting this out. We have corrected the typos in the revised version of the paper.

The authors included a reference to source code that could be used to reproduce the results, and this is lauded by the reviewer. The reviewer downloaded the code and ran the experiment in it locally and attained comparable results. The reviewer used 20 epochs for all of the following mentioned experiments. With five trials, the reviewer received a 96.804% (+/- 0.094) mean accuracy using the code published by the authors.

The batch size of 5,000 is unusually large, so the reviewer repeated the experiment with a batch size of 100 and achieved a 97.524% (+/- 0.044) mean accuracy. The authors would be advised to use the smaller batch size in the revision to attain better results.

We thank the referee for this suggestion. We have reduced the batch size in our new experiments. Also we have changed the non linearity to a ReLU activation.

The reviewer chose to implement a potential baseline experiment by replacing the spectral layers with traditional fully connected layers with batch normalization and ReLU activation (this is the current standard/common practice). The reviewer used the same number of layers (3) and chose the neurons so that the network had a nearly identical number of trainable parameters. The authors can replace these lines in their source code:

```
model.add(SpectralLayer(500,is_base_trainable=False,activation='tanh'))
model.add(SpectralLayer(200,is_base_trainable=True,activation='tanh'))
model.add(SpectralLayer(10,is_base_trainable=True,activation='softmax'))
```

with these to get the same baseline:

```
model.add(tf.keras.layers.Dense(120))
model.add(tf.keras.layers.BatchNormalization())
model.add(tf.keras.layers.Activation('relu'))
model.add(tf.keras.layers.Dense(60))
model.add(tf.keras.layers.BatchNormalization())
model.add(tf.keras.layers.Activation('relu'))
model.add(tf.keras.layers.Dense(10, activation='softmax'))
```

When using a batch size of 5,000, this baseline achieved a 97.054% (+/- 0.161) mean accuracy. With a batch size of 100, a 98.058% (+/- 0.085) mean accuracy was achieved.

The reviewer then went on to experiment with a 3-layer convolutional neural network with a similar number of parameters. The relevant code follows (the shaping of the input data is omitted):

```
model.add(tf.keras.layers.Conv2D(12, (3, 3)))
model.add(tf.keras.layers.BatchNormalization())
model.add(tf.keras.layers.Activation('relu'))
model.add(tf.keras.layers.Conv2D(16, (3, 3)))
model.add(tf.keras.layers.BatchNormalization())
model.add(tf.keras.layers.Activation('relu'))
model.add(tf.keras.layers.Conv2D(20, (3, 3)))
model.add(tf.keras.layers.BatchNormalization())
model.add(tf.keras.layers.Activation('relu'))
model.add(tf.keras.layers.Flatten())
model.add(tf.keras.layers.Dense(10, activation='softmax'))
```

When using a batch size of 100, this achieved a 98.826% (+/- 0.148) mean accuracy.
The authors would be well advised to put forward such baselines and work towards showing their method as superior.

We thank the referee for exploring this setting. As suggested, we have reduced the batch size and adopted the ReLU filter. We however prefer not to involve the convolutional layers in this first application, as a fair comparison would require integrating spectral schemes and convolutional techniques. As illustrated above, the idea here is to set the stage for the spectral approach, within the most basic framework for deep learning.

Answer to Reviewer #4

The manuscript "Machine learning in spectral domain" is sound and mostly well written.
Although it contains some interesting ideas, I fail to see any particularly novel result or strong conceptual/computational advance supporting its publication on Nat. Comm. Also, comparison with related literature and benchmarking against models therein is missing, as is also missing an in-depth assessment of the performance of the proposed model. As the paper currently stands, the authors fail to substantiate the claim that the proposed paradigm is a promising alternative for deep learning practice or theory, but I do not exclude the possibility of changing my mind if much stronger evidence is provided.

We thank the referee for the overall positive evaluation of our manuscript. As mentioned above, we are confident that anchoring the training to reciprocal space could translate in a novel approach to supervised learning, alternative and complementary to existing strategies. In the attempt to clarify the reference

conceptual framework, we elaborate further on the simplified setting where eigenvalues are solely optimized. More specifically, we now show that by tuning the eigenvalues, while freezing the eigenvectors, yields performances which are superior to those attained with usual algorithms restricted to operate with an identical number of free parameters. Further, spectral learning bound to operate on the eigenvalues returns a distribution of the optimized weights which is close to that obtained when training the neural network in direct space, with no restrictions on the parameters to be tuned. Eigenvalues are thus parameters of key importance for neural networks training, way more strategic than any other set of identical cardinality in the space of the nodes. Spectral learning acts globally (changing e.g. the eigenvalues amounts to modulate simultaneously all the weights in direct space), at variance with conventional strategies which seek at implementing local moves in the space of the nodes. This point is now raised in the revised version of the manuscript. Different settings are explored and the obtained results displayed in a novel set of figures which we hope will help clarifying the overall message.

- This is easily fixed, but caused me some major pain in understanding the paper: it is not made explicit how to construct vec{n} beyond the first N_1 components. I assume the last N-N1 components to be just set to 0, but really, this should be clearly stated.
The same goes for the entries of the base vectors \phi_k, which I assume to take value 0 except for the diagonal and the sub-diagonal blocks.

We apologize for this. In the revised version of the paper we have entirely rewritten the part devoted to introducing the mathematical setting and clarified the definition of the involved quantities.

- The framework boils down to a standard deep network where each layer performs a linear transformation of the input, possibly followed by an elementwise non-linearity. The only difference with the standard approach is in the structured form of the weight matrices, depending quadratically on the eigenvectors phi and linearly on the eigenvalues lambda.

As the referee correctly recognizes, when framing the problem in the space of the nodes, one recovers a standard neural deep network, with a feed-forward architecture. This reflects the specific structure of the eigenvectors that we have chosen to deal with. Our aim is in fact to show that standard networks can be trained via a different algorithmic approach, which is made possible by representing the learning process in dual space. However, the proposed method is not limited to the explored setting, which bears pedagogical interest. By assuming other **non-orthogonal** basis will allow in turn to inspect other scenarios and recover different architecture in direct space (see also below).

Non-orthogonality of the eigenvectors is indeed the key ingredient for an effective information processing, from the input to the output. This issue is now brought up in the revised version of the paper.

The number of training parameters is reduced only when fixing the vectors phi, with subsequent severe performance impact. No time or memory gain is obtained in either case (actually, it could quite be the contrary).

Our aim was to show that the eigenvalues constitute a set of key importance, and this becomes transparent only when carrying out the training in dual space. In the revised version of the paper we have included a set of Figures to report on several novel tests that we have performed to hopefully clarify the ideas underlying the spectral approach to the learning. As an example, we find particular intriguing the observation that restricting the learning to the eigenvalues yields a distribution of the weights which resembles quite closely that obtained in direct space when performing the training on a much larger set of parameters.

I can think of a few approaches that involve learning structured matrices, some of which also lead to time and space complexity gains:
- separable convolutions
- low-rank factorizations, as in
"Low-rank matrix factorization for Deep Neural Network training with high-dimensional output targets", DOI:10.1109/ICASSP.2013.6638949
- training of singular values of the SVD decomposition only, as discussed in
"Entropy and mutual information in models of deep neural networks" DOI:10.1088/1742-5468/ab3430
- porcupine neural networks and related models:
https://arxiv.org/abs/1710.02196
https://arxiv.org/abs/2003.00152

None of these approaches is discussed in the manuscript, let alone compared and benchmarked against the proposed model.

When training only lambdas instead, it would be interesting to see a comparison with the technique of
https://arxiv.org/abs/2003.00152
or
"Entropy and mutual information in models of deep neural networks" DOI:10.1088/1742-5468/ab3430

We thank the referee for bringing these papers to our attention. In the revised version of the manuscript, we now acknowledge the similarities with other

existing methods, as suggested. However we do not carry out a systematic comparison in terms of achieved performance, as this is not the goal of our work, which aims instead at a new proof-of-concept. We are not claiming that our method outperforms other schemes but that it opens up interesting, so far unexplored possibilities, that we try, at least partially, to critically address. It should also be remarked that none of the papers mentioned seem to tie the learning to the spectral domain.

- When also training the eigenvectors phi_k, the only reduction in the number of trained parameters comes from freezing the first layer.
In this setting, performance should be compared against standard deep networks with the same number of trained and untrained parameters where the first layer is frozen.

In fact, the claim "Learning on the eigenvalues and eigenvectors allows to significantly reduce the number of free parameters usually managed in conventional neural networks" should be validated comparing with standard architectures with the same number of trained AND untrained parameters, while only a fully trained standard network with no hidden layers was used for the result in fig. 3.
As a side note, since this setting is also referred to as "extreme learning", the authors should
mention and comment on this.

We thank the referee for this constructive criticism. In the revised version of the paper we now make comparison with the results obtained with baseline architectures operated with a similar number of trained parameters. We show in particular that by adjusting the eigenvalues, when freezing the eigenvectors entries, yields classification scores which are superior than those obtained with usual algorithms restricted to operate with an identical number of free parameters. The claims have been thoroughly revised and the text modified so as to reflect the quantitative conclusions that we have reached with the newly added tests.

In extreme learning the parameters of the hidden nodes (and not just the weights connecting inputs to hidden nodes) need not be tuned. In our setting, we adjust a limited number of parameters, e.g. the eigenvalues, which however drive a (global) modulation of all the weights in direct space.

- Practical implementation and computational complexity of the algorithm are not clearly stated.
I assume that a reasonable implementation of a spectral layer involves multiplication with (N_k + N_{k+1}) x (N_k + N_{k+1}) matrices in the forward

pass, so roughly twice the size of a corresponding standard layer. Could this be brought down to multiplication of N_k x N_{k+1} matrices exploiting (again) the block structure?

In the revised version of the manuscript we have added a paragraph to discuss the actual implementation of the algorithm. The gap between layers (k) and (k+1), is bridged by a matrix of dimensions N_k x N_{k+1}, as correctly stated by the referee. The peculiar form of the imposed eigenvectors makes it possible to analytically access the inverse of \Phi, with significant computational advantages. This is instead no longer the case if one deals with other non-orthogonal basis. We recall that this could be the case when aiming at exploring other settings in direct space, alternative to a standard feedforward architecture.

- I may be wrong, but I don't think that statements as "It is in fact tempting to surmise that the
nested arrangement of the eigenvectors of the adjacency matrix which define the network in
direct space is a key property for the neural network to carry out the assigned classification
task." or "By formulating the learning process in reciprocal
space, we have isolated an important aspect which, we believe, could form the basis for a
rational understanding of the surprising ability of deep networks to cope with the assigned
tasks" have been really substantiated, and that the so-called "nested structure" brings
any more understanding or is conceptually any different from the plain concept of forward propagation.

The text has been thoroughly revised and the above sentences removed. Our intention was to communicate the following message. Working in the spectral domain, we show that the information is passed between interlaced eigendirections. For a feed-forward setting in direct space, the indentation (as we call it) of the eigenvectors associated to different layers is a rather obvious requisite to assume. The proposed method is however way more general and not solely limited to the setting that we have explored for pedagogical reasons. It can in fact generalize to other frameworks, provided one deals with a non orthogonal basis (hence indented) in reciprocal space. For this reason, we speculated, in the previous version of the manuscript, that nested indentation represents a universal paradigm. We however agree with the referee that this was not sufficiently motivated. In the revised version of the manuscript we discuss some aspects related to this possibility, and emphasise the key role played by non-orthogonality.

- Number of parameters in ordinary schemes do not necessarily ramp up as N^2 as stated in the conclusions, there is an interplay between depth d and width w,
*number of parameters being w^2 * d and N = w*d.*

We agree and we have corrected this throughout the manuscript.

Reviewer #1 (Remarks to the Author):

This revision is much improved and well addressed my initial comments in most cases.

In the introduction, the improvements include (1) removing unsubstantiated claims and vagueries, and (2) correcting inexact language related to AI and the usual description of the processes involved in deep learning.

The experimentation is much improved as well.

The experiments are entirely reproducible and the code is readily available, which is a strength for this line of research.

At this point, I am now able to say there are no serious problems that give me pause, and the criticisms that follow are not meant to be taken as reasons for rejection, but rather as advice on how to further improve the paper and as a result its potential impact.

There are still several places where the English is less than perfect. I would encourage the authors to use an advanced grammar checker like Grammarly. Examples:
(1) "...restricted to a operate with an identical..." The indefinite article "a" does not belong there.
(2) "These blocks yields an effective..." The plural of the subject "blocks" does not comport with the singular verb "yields".
(3) "...one can imagine to recast the whole..." "to recast" should not be rendered as an infinite, but rather as "recasting"

The Results section could be better organized to make the comparisons that are now present easier to understand. Specifically, tables would improve this considerably.
Additional experiments performed on other small image datasets like Fashion-MNIST, CIFAR-10, and CIFAR-100, along with the results of them in the suggested tables would benefit this paper. Noting that the experiments on the additional datasets wouldn't need to exhaustively elucidated.

Signed,
Adam Byerly

Reviewer #4 (Remarks to the Author):

The manuscript has greatly improved after the revision,
and I find its results interesting and a good contribution to the literature.
Unfortunately, there is no convincing evidence in support
of the conceptual or practical utility of the framework proposed,
therefore I cannot recommend the manuscript for publication in Nature Communications.

Some further comments:

- Only one experiment for the full training of eigenvalues and eigenvectors is reported, on a network with no hidden layers. Reperted accuracy is on par with that of the standard network. Assuming the same holds true for deeper architecture, no gain is acquired despite additional implementation and computational complexity.

- In ref. 22, a similar spectral training was proposed, i.e. training only the singular values of the singular value decomposition of the weight matrices. The authors should

compare the two algorithms.

- The authors stress the relevance of their "global" training as
opposed to the "local" training in standard networks.
I don't think the is any particular shift in paradigm if one considers
that one of their layers effectively corresponds to a 2 layer network with tied weights and additional per-neuron parameters. Is training per-neuron parameters
any more global than training weights?

- The authors provide evidence that training the eigenvalues only is
better than training a random subset of the weights of the same size.
In my opinion, this is not enough to substantiate the claim:
"Eigenvalues are therefore identified as key target of the learning process,
proving more fundamental than any other set of identical cardinality, allocated
in direct space."
At the very least, the authors should compare against some more structured
subset training, e.g. Ref. 21 (see also the comment above).

- Eq. 8 corresponds to a MSE loss applied to the output of a softmax layer.
Right below the equation, the authors say they use a cross-entropy loss
in their experiments. It is not clear at this point if they are really using a cross-entropy loss or equation 8 as their loss.

Answer to reviewer #1

This revision is much improved and well addressed my initial comments in most cases. In the introduction, the improvements include (1) removing unsubstantiated claims and vagueries, and (2) correcting inexact language related to AI and the usual description of the processes involved in deep learning. The experimentation is much improved as well. The experiments are entirely reproducible and the code is readily available, which is a strength for this line of research.
At this point, I am now able to say there are no serious problems that give me pause, and the criticisms that follow are not meant to be taken as reasons for rejection, but rather as advice on how to further improve the paper and as a result its potential impact.

We thank the referee for the positive evaluation of our paper and for deeming it worth publication. In the following we provide our reply to the remarks and suggestions for improvement.

There are still several places where the English is less than perfect. I would encourage the authors to use an advanced grammar checker like Grammarly. Examples:
(1) "...restricted to a operate with an identical..." The indefinite article "a" does not belong there.
(2) "These blocks yields an effective..." The plural of the subject "blocks" does not comport with the singular verb "yields".
(3) "...one can imagine to recast the whole..." "to recast" should not be rendered as an infinite, but rather as "recasting"

We apologize for these mistakes. We have corrected the above errors and carefully checked the manuscript with a grammar checker.

The Results section could be better organized to make the comparisons that are now present easier to understand. Specifically, tables would improve this considerably.

We have inserted a table in the Results section to make the outcome of the analysis immediately accessible.

Additional experiments performed on other small image datasets like Fashion-MNIST, CIFAR-10, and CIFAR-100, along with the results of them in the suggested tables would benefit this paper. Noting that the experiments on the additional datasets wouldn't need to exhaustively elucidated.

Following the advice of the referee, we have added a Supplementary information to illustrate the results obtained for the Fashion-MNIST database. We decided instead not to include the results obtained with CIFAR-10 and CIFAR-100. In the tests that we performed on CIFAR-10 and CIFAR-100, training the eigenvalues within a simple feedforward architecture allows one to achieve better performance as compared to those obtained when anchoring the training in direct space and using an identical number of free parameters. Stated differently the results are in line with those obtained for NMIST and

Fashion-MNIST. However, simple feedforward networks applied to CIFAR-10 and CIFAR-100 allow one to reach an accuracy of at most 60% on the test set. Convolutional layers need to be introduced for enhancing the performance of the classification scheme and this holds true for both direct and spectral learning. We prefer however to postpone to a future contribution the problem of addressing the efficient integration of convolutional layers and spectral learning. We believe in fact that this could be dealt more efficiently (in terms of achieved gain) than the obvious solution of appending spectral feedforward layers after the convolutional ones but this issue deserves to be further explored.

Answer reviewer #4 (Remarks to the Author):

The manuscript has greatly improved after the revision, and I find its results interesting and a good contribution to the literature. Unfortunately, there is no convincing evidence in support of the conceptual or practical utility of the framework proposed, therefore I cannot recommend the manuscript for publication in Nature Communications.

We thank the referee for her/his appreciation of the revised manuscript and for judging the results interesting. In the following we provide a detailed answer to the raised issues.

- Only one experiment for the full training of eigenvalues and eigenvectors is reported, on a network with no hidden layers. Reported accuracy is on par with that of the standard network. Assuming the same holds true for deeper architecture, no gain is acquired despite additional implementation and computational complexity.

In Figure 7 the performance of the method is displayed when both eigenvectors and eigenvalues are simultaneously trained and for a feedforward neural network with two hidden layers. Additional tests are now supplied as supplementary information. As also stated in our first rebuttal letter, we do not aim at challenging state-of-the-art implementations in the competition for the best classification performance of the MNIST database. Our goal is to propose a proof-of-concept of a novel route to supervised learning.

- In ref. 22, a similar spectral training was proposed, i.e. training only the singular values of the singular value decomposition of the weight matrices. The authors should compare the two algorithms.

In Ref 22 the authors propose a Single value decomposition of W_k, the rectangular matrix of the weights that link two adjacent layers (resp. k and k+1). In formulae W_k=U_k S_k V*_k. The columns of matrix U_k and the rows of matrix V*_k are the eigenvectors of, respectively, W_k W*_k and W*_k W_k. Matrix S_k is diagonal and it contains the singular values W_k. The singular values are related to the eigenvalues of W_k W*_k and W*_k W_k, square matrices constructed from W_k. In our setting, the problem is reformulated in a space of extended dimensions which enables one to deal with square matrices and so invoke the spectral theorem. The eigenvalues are hence stemming from the adjacency

matrix that sets the architecture of the underlying neural network, and, as such, they bear a clear interpretation. The eigenvalues gauge in fact the importance of the associated eigendirections, veritable avenues where information is eventually transported between successive stacks of the directed network arrangement. Tuning the eigenvectors imply, in our setting, optimizing the routes in reciprocal space where the information flows. In our view, the similitude of the two approaches solely resides in the idea of highlighting a (different) subset of parameters (which total in N, the total number of neurons) to be optimized. Our analysis is specifically aimed at implementing and testing an efficient learning scheme anchored to reciprocal space (which we challenge against standard approaches to the learning), while the focus of Ref. 22 is on testing the behavior of entropies and mutual information throughout learning. In the revised version of the manuscript we have inserted a sentence to reflect the above points.

- The authors stress the relevance of their "global" training as opposed to the "local" training in standard networks.
I don't think the is any particular shift in paradigm if one considers that one of their layers effectively corresponds to a 2 layer network with tied weights and additional per-neuron parameters. Is training per-neuron parameters any more global than training weights?

By slightly adjusting individual eigenvalues yields a global rearrangement of all the weights which are customarily trained in direct space. This is why we point to the global effect produced in direct space by local moves acting in the spectral domain.

- The authors provide evidence that training the eigenvalues only is better than training a random subset of the weights of the same size. In my opinion, this is not enough to substantiate the claim: "Eigenvalues are therefore identified as key target of the learning process, proving more fundamental than any other set of identical cardinality, allocated in direct space." At the very least, the authors should compare against some more structured subset training, e.g. Ref. 21 (see also the comment above).

Following the referee remark we have slightly rephrased the above sentence to reflect the fact that the subset of trainable weights is randomly selected.

- Eq. 8 corresponds to a MSE loss applied to the output of a softmax layer. Right below the equation, the authors say they use a cross-entropy loss in their experiments. It is not clear at this point if they are really using a cross-entropy loss or equation 8 as their loss.

Eq. (8) is introduced, in analogy with eq. (7), for illustrative purposes (as stated just before eq. 7). In the actual implementation we made use of a cross entropy loss, in both settings (linear and nonlinear) explored within the paper.
